# Radiation-induced bystander and abscopal effects: important lessons from preclinical models

**DOI:** 10.1038/s41416-020-0942-3

**Published:** 2020-06-25

**Authors:** Elisabeth Daguenet, Safa Louati, Anne-Sophie Wozny, Nicolas Vial, Mathilde Gras, Jean-Baptiste Guy, Alexis Vallard, Claire Rodriguez-Lafrasse, Nicolas Magné

**Affiliations:** 1 Département de Radiothérapie, Institut de Cancérologie Lucien Neuwirth, Saint-Priest-en-Jarez, France; 2 Département Universitaire de la Recherche et de l’Enseignement, Institut de Cancérologie Lucien Neuwirth, Saint-Priest-en-Jarez, France; 3 Université de Lyon 1, UMR CNRS5822/IN2P3, IP2I, PRISME, Radiobiologie Cellulaire et Moléculaire, Faculté de médecine Lyon-Sud, Oullins Cedex, France; 4 Hospices Civils de Lyon, Centre Hospitalier Lyon-Sud, Pierre-Bénite, France

**Keywords:** Tumour immunology, Molecular medicine

## Abstract

Radiotherapy is a pivotal component in the curative treatment of patients with localised cancer and isolated metastasis, as well as being used as a palliative strategy for patients with disseminated disease. The clinical efficacy of radiotherapy has traditionally been attributed to the local effects of ionising radiation, which induces cell death by directly and indirectly inducing DNA damage, but substantial work has uncovered an unexpected and dual relationship between tumour irradiation and the host immune system. In clinical practice, it is, therefore, tempting to tailor immunotherapies with radiotherapy in order to synergise innate and adaptive immunity against cancer cells, as well as to bypass immune tolerance and exhaustion, with the aim of facilitating tumour regression. However, our understanding of how radiation impacts on immune system activation is still in its early stages, and concerns and challenges regarding therapeutic applications still need to be overcome. With the increasing use of immunotherapy and its common combination with ionising radiation, this review briefly delineates current knowledge about the non-targeted effects of radiotherapy, and aims to provide insights, at the preclinical level, into the mechanisms that are involved with the potential to yield clinically relevant combinatorial approaches of radiotherapy and immunotherapy.

## Background

Up until the past 10 years, it was generally accepted that the effects of radiotherapy were mediated by direct damage to DNA or from indirect damage through free radicals generated by water radiolysis. Indeed, early radiobiological studies reported that the major mechanisms of action of ionising radiation were related to DNA damage.^1,2^ However, this assumption has been challenged by numerous observations showing that non-irradiated cells either nearby or located away from the site of irradiation can sometimes undergo the same response as irradiated cells,^3–6^ and it has subsequently been discovered that cancer cells subjected to ionising radiation can release signals that are able to influence the outcome of non-irradiated cells.^7^

We can distinguish three types of non-targeted effect, which depend on the relationship between the irradiated and non-irradiated cell, as well as the proximity to the original site of treatment.^4^ The extent of these effects, in terms of distance, has not been formally determined and might differ when studied in vitro or in vivo. According to the United Nations Scientific Committee on the Effects of Atomic Radiation (UNSCEAR), the radiation-induced bystander effect is a radiobiological effect that is transmitted from irradiated cells to neighbouring unirradiated cells, leading to biological changes in the recipient cells.^8,9^ The radiation-induced abscopal effect (from the Latin ‘ab scopus’, meaning ‘away from the target’) is a local radiation-induced systemic effect that extends outside the treated volume, and is able to drive the regression and rejection of non-irradiated, distant tumour lesions.^10^ Although no fixed margin of the effects can necessarily be defined, a topographical distinction can be made between these two non-targeted effects. The bystander effect could simply be described as a local communicative effect at the primary site over a few millimetres or cellular diameters, mediated through the secretion of soluble factors or by signalling through gap junctions as well as through networks involving inflammatory cells of the microenvironment. By contrast, the abscopal effect is a long-distance (up to tens of centimetres outside of the irradiated field) and systemic effect at a distant metastatic site that is mediated through immunogenic responses.^6,11,12^ In the context of irradiated/non-irradiated sites within the same organ, it remains difficult to distinguish between a long-range bystander effect and an abscopal effect. Although less well investigated, a third type of non-targeted effect—the cohort effect—is defined as the interaction between irradiated cells within an irradiated volume where, under heterogeneous irradiation, high-dose-irradiated cells might affect low-dose-irradiated cells, and vice versa;^13^ the cohort effect is limited to an area of millimetres within the target.^14^

**Fig. 1:**
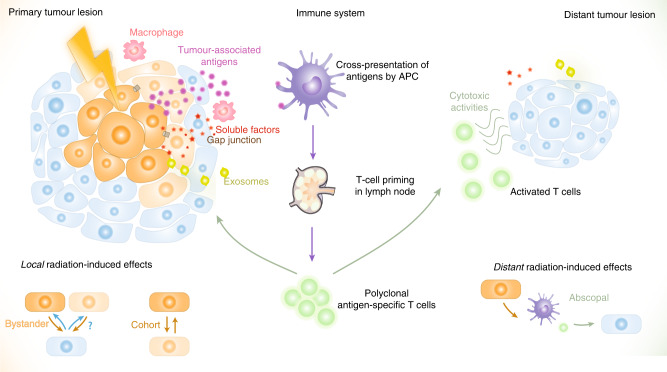
Schematic overview of local and distant effects triggered by tumour irradiation. At the heart of the primary lesion that is irradiated (panel on the left), two local effects can be distinguished: first, bystander effects occur between high-dose-targeted cells (dark orange) or low-dose-targeted cells (light orange) and non-irradiated cells (blue); second, cohort effects occur between high-dose-targeted cells and low-dose-targeted cells. Whether/how non-irradiated cells can influence the outcome of irradiated cells (depicted with a question mark) remains to be determined. Irradiation induces immunogenic cell death in cancer cells and the subsequent release of tumour-associated antigens (TAAs) (pink dots), thereby activating the immune system, especially antigen-presenting cells (APC, in purple) and macrophages (in pink). APCs then cross-present TAAs to T cells in draining lymph nodes. As a result, polyclonal antigen-specific T cells are primed to attack tumours located within the irradiated field as well as those in distant locations. This distant radiation-induced effect is termed an abscopal effect (panel on the right). Exosomes (in green) are novel mediators thought to participate in these non-targeted effects locally and at distant sites.

Research is still ongoing to unravel the molecular pathways that underlie these effects, and to transfer these discoveries to innovative clinical applications.^15^ Numerous key mediators have been considered responsible for the bystander effect and the abscopal effect. However, as the scope of this review is beyond describing the features of each effect in detail or detailing the interplay between radiation and immunity, we invite readers to refer to other comprehensive reviews on this topic (for reviews see refs. ^12,14–20^). Briefly, though, the main mechanism responsible for the bystander effect involves direct gap-junction-mediated cell–cell communication via ions such as calcium and small molecules such as nitric oxide, although other factors such as transforming growth factor-β (TGF-β), cytokines and chemokines can also be released in the extracellular compartment, triggering local immune activation (Fig. 1, left panel). Interestingly, a systemic long-distance abscopal effect arises if this non-specific inflammatory-type response reaches distant locations,^12^ although the abscopal effect is generally thought to be mediated by T-cell activation. In addition to the direct secretion of cytokines and chemokines, exosomes have been identified as novel systemic mediators of communication between irradiated and non-irradiated cells or tissues, and might therefore be involved in the bystander, abscopal and cohort effects. The nature of their cargo is a matter of intensive research, but might include microRNAs, long non-coding RNAs, protein mediators, immune players, etc.^21^ With regard to the abscopal effect, the involvement of the immune response in this phenomenon can be outlined by a simplified scenario (Fig. 1, right panel). Tumour cells undergoing immunogenic forms of cell death in response to ionising radiation are known to release danger signals and damage-associated molecular patterns (DAMPs), and to expose tumour-associated antigens (TAAs), which can attract or activate immune-related cells.^16,22–25^ Radiotherapy is known to modulate several aspects of a variety of immune-related components from the tumour ecosystem: the expression of TAAs on tumour cells,^26,27^ the expression on endothelial cells of adhesion molecules for leukocyte recruitment,^28–30^ the nature of antigen-presenting cells (APCs) (e.g. the maturation of dendritic cells, increased expression of major histocompatibility complex class I molecules, increased peptide repertoire and cross-presentation and increased expression of co-stimulating molecules),^27,31^ the behaviour of cytotoxic T lymphocytes (CTLs) (e.g. priming and activation of T cells, cell death of suppressor T cells),^32,33^ and the secretion of cytokines and chemokines as signalling orchestrators for adequate systemic activation of the immune system.^34,35^ Hence, irradiated tumours are often referred to as ‘in situ vaccinations’ that supply TAAs to APCs, which then cross-present these species to T cells. As a result, the migration of effector T cells from lymph nodes to distant tumour sites to mediate tumour-cell destruction is stimulated^36,37^ (Fig. 1). Consequently, it is well accepted that immune mechanisms are the driving forces of such responses.

Early reports of the abscopal effect in response to radiation alone were rare, however. Because of its low incidence (46 clinical cases from 1969 to 2014), not only did the radiotherapy-related abscopal effect arouse great interest, but also scepticism, as it remained sporadic.^38^ This rare occurrence can be explained by the radiotherapy-mediated attraction of immunosuppressive cells into the tumour microenvironment as a self-protective mechanism of radioresistance, which thereby inhibits T-cell priming. A combined approach with immunotherapy, however, has led to an increase in the occurrence of the abscopal effect,^39,40^ and appears important in overcoming the barriers and synergising the efficacy of each intervention alone.^41,42^

Although several preclinical studies have helped to understand how the combined approach of immunoradiotherapy might potentiate the systemic control of tumorigenesis, there is still much work to do in order to increase our knowledge about radiobiology and investigate the discrepancies between preclinical models and clinical experiences. The aim of this review is to propose research perspectives that might be relevant to narrow the translational bridge from bench to bedside.

## The bystander effect

Cells that undergo a radiation-induced bystander effect demonstrate reduced clonogenic survival, increased sister chromatid exchange, the formation of micronuclei and apoptosis and altered gene expression and levels of RNA transcripts^43^ in response to molecular signals transmitted from irradiated cells through direct contact (e.g. through intercellular gap junctions) or through the release of diffusible factors such as cytokines and chemokines.^20,43,44^ As mentioned above, exosomes have also been identified as novel systemic communication mediators between irradiated and non-irradiated cells or tissues. Immune-related phenomena are involved in mediating the bystander effect via the secretion of inflammatory mediators, interferons (IFNs) and appropriate chemokines, which attract T cells. Moreover, radiotherapy might enhance T-cell trafficking to primary tumours through local vascular endothelial inflammation.^45^ Macrophages are also well-known players in the bystander signalling cascade in which, once activated by irradiated cells, they further damage other neighbouring cells by transferring bystander signalling factors.^46–48^ Notably, not only do bystander cells respond to signals from irradiated cells, but they can also influence irradiated cells, thereby suggesting the existence of a reciprocal dialogue that modulates the responses to ionising radiation.^49^ In some cases, bystander signalling can confer increased survival on non-irradiated cells.^50^

### Current in vitro systems for investigating the bystander effect

The multiple radiation-induced bystander effects are likely to be influenced by biological characteristics, such as the nature of the irradiated cell type, the nature of the bystander cells and the composition of the intercellular milieu, as well as the physical parameters of radiotherapy (the dose, rate, type of irradiation and time after exposure). Accordingly, in vitro systems have been developed to further investigate the radiation-induced bystander effect. These approaches include conventional irradiation of monolayer cell cultures with low doses, cell culture medium transfer experiments (in which a cell culture medium is harvested from irradiated cells and used to treat unexposed cells), partial shielding of monolayer cultures during irradiation (half-shielded dishes, or using grid or slit irradiation), co-culture irradiation systems (in which irradiated cells are grown as a monolayer, while non-irradiated cells are cultured in a Transwell chamber with a porous membrane, allowing factors to diffuse between the two compartments) and the use of microbeams to directly set amounts of irradiation to precise locations in two-dimensional (2D) cell cultures.^51^ However, the extracellular environment of rapidly expanding cells might not be representative of the tissue environment and, consequently, several three-dimensional (3D) models from various origins (e.g. skin, endothelium, breast and lung) have been established and exposed to low or high linear energy transfer (LET) radiation with partial shielding.^52–58^ Using these different models, the effects of ionising radiation have been mostly elucidated at the cellular (e.g. compromised proliferation and cell-cycle progression, apoptosis) and molecular levels (e.g. γ-H2A histone family member X [γ-H2AX] and p53-binding protein 1 [53BP1] as markers of DNA damage foci, secretion of factors such as inflammatory cytokines). However, the identity and regulation of the molecular dynamics of the processes require further investigation.

### Using 2D cultures without cellular contact to increase our molecular knowledge of bystander and cohort effects

To date, no study has systematically mapped gene expression changes during the bystander transition using deep-sequencing technologies such as RNA sequencing. So far, global gene expression has been assessed in cells treated with conditioned media from irradiated cells or in co-culture (for review see ref. ^59^) using microarray chips, but the coverage is too low, and the results do not reflect the magnitude of the effects, rendering this technology effectively obsolete. Furthermore, the effects of irradiated cells on non-irradiated cells and vice versa also require investigation. To identify this bidirectional interaction, it would be interesting to comparatively interrogate the RNA landscape of each population of cells, both in conditions of co-culture and separately. This analysis would be informative at multiple levels. First, it would reveal changes induced by irradiation on non-irradiated cells. Second, it would identify any changes induced by non-irradiated cells on irradiated cells. Furthermore, it would enable a genome-wide map of gene expression, including protein-coding genes (mRNAs) and non-coding genes (long non-coding RNAs, small nucleolar RNAs and small nuclear RNAs) to be generated. Splicing changes and splice variants, as a read-out of protein isoforms, could also be analysed. Importantly, membrane-anchored proteins that could potentially be targeted by immunotherapy or act as potential biomarkers of immunotherapy response could be identified.

**Fig. 2:**
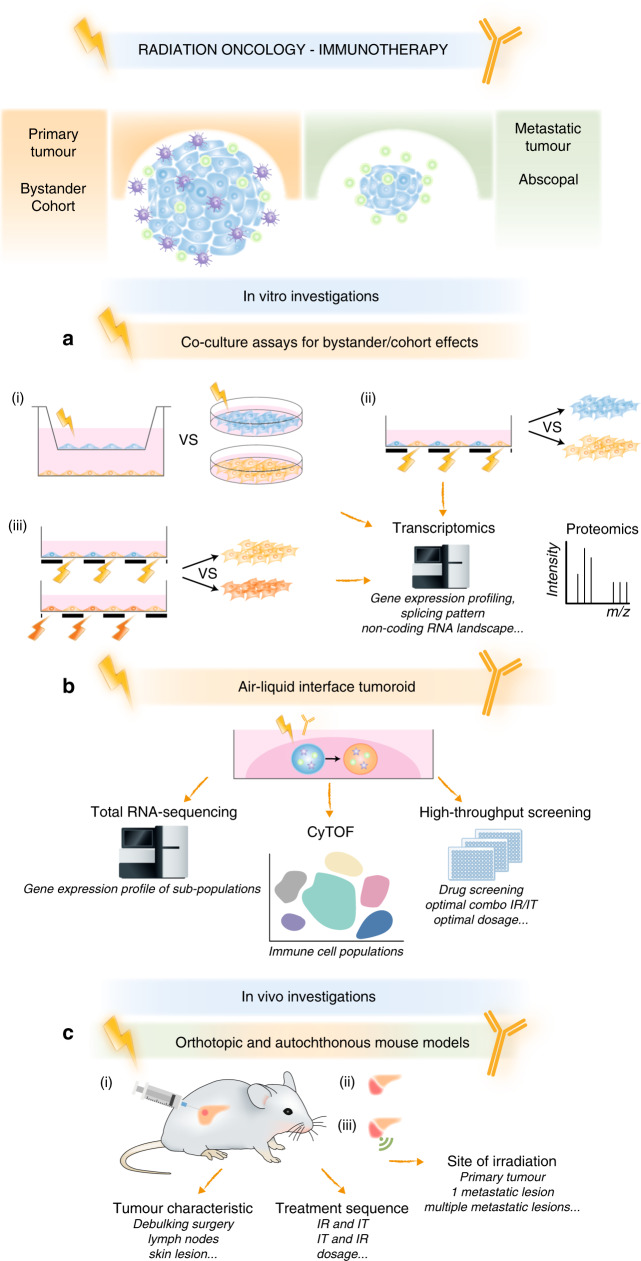
Preclinical experimental strategies for efficient radio-immunotherapy combinations in clinical routine. **a** In vitro investigations using 2D cell cultures and next-generation sequencing: (i) comparative analysis between co-culture assays without contact and solely monocultures (one irradiated [blue] and one non-irradiated [dark orange]); (ii) co-culture assays with contact using partial irradiation through grids (black boxes) and following cell sorting; (iii) co-culture assays with contact using partial irradiation through grids and with two doses (yellow and orange ‘thunders’). **b** In vitro investigations using 3D tumour models (in blue) containing endogenous immune cells (antigen-presenting cells [purple stars], lymphocytes [green circles]) for testing the radio-immunotherapy combination, in order to decipher the molecular mechanisms by RNA sequencing, to identify the immune repertoire by mass cytometry (CyTOF) and to develop high-throughput technologies for drug screening, treatment schemes, etc. **c** In vivo investigations relying on (i) orthotopic, (ii) local autochthonous and (iii) disseminated autochthonous tumour mouse models for testing the radio-immunotherapy combination.

Monitoring the secretome of both cellular compartments using conditioned media might also provide insight into the bystander effect. Mass spectrometry or enzyme-linked immunosorbent assay (ELISA) techniques have been used to identify diffusible factors secreted by irradiated cells (e.g. TGF-β, tumour necrosis factor-α [TNF-α], interleukin [IL]-8, IL-1 and vascular endothelial growth factor [VEGF]), but those secreted by bystander cells remain largely unidentified. MS analyses of the co-culture medium do not enable the source of factors to be distinguished, and ELISA techniques rely on candidate-based approaches, but RNA sequencing might provide clues regarding potential molecules and signalling pathways (Fig. 2a). The development of powerful quantitative proteomics approaches such as spike-in SILAC (for Stable Isotope Labelling by Amino Acids in Cell culture) or triple SILAC^60^ might facilitate the analysis of the differential proteomes (secretomes and intercellular proteins) in irradiated cells and neighbouring cells (Fig. 2a).

### Using 2D cultures with cellular contact to increase our molecular knowledge of bystander and cohort effects

To decipher the importance of cell contacts in the bystander and cohort effects, co-culture assays that use cell-patterning procedures are required. A contact-erasing system—termed µ-eraser strategy—has been engineered to fabricate a co-culture micropattern arrangement, which involves pressing a stamp to induce cell lysis and then seeding another type of cell on the empty regions.^61^ The incorporation of a fluorescent lipophilic dye into one of the cell types would then make it possible to seed two cellularly distinguishable populations. Non-labelled cells could be specifically irradiated using partial shielding with a grid that resembles the original cell pattern. Following irradiation and trypsinisation, non-labelled irradiated and labelled non-irradiated cells could be separated using flow cytometry for subsequent comparative transcriptomic or proteomic analysis. Clustering analysis would then enable pathways and/or mediators related to cellular contacts or to paracrine effects to be connected, and the mutual crosstalk induced by ionising radiation to be dissected. Similar transcriptomic and proteomic approaches could be applied to decode cohort effects: here, micropatterned cells would be irradiated with two different doses, following specific partial shielding and flow cytometry clustering of both populations (Fig. 2a).

### Using 3D cultures to increase our molecular knowledge of bystander and cohort effects

Although in vitro settings are considered to be a highly controlled artificial environment, they do represent a simplified system that is useful for deconstructing primary molecular events. However, the principal limitations of the cell culture models described so far are the existence of only two dimensions and the absence of physiological features, especially the microenvironment and its immune compartment, reinforcing the notion that these models cannot be regarded to biologically mimic tumours. As it is well known, the tumour microenvironment crucially influences the response to treatment. Furthermore, there is no doubt that both stromal cells and the immune system can contribute to, or inhibit, tumour eradication after irradiation. 3D organoid structures can be supplemented with a sham reconstitution of the microenvironment by co-culturing with peripheral blood lymphocytes or tumour-infiltrating lymphocytes (TILs), as well as by adding stromal components, such as extracellular matrix or fibroblasts.^62–66^ However, such additions cannot recapitulate the physical architecture of the tumour microenvironment, due to the diversity of immune cells and the presence of other non-immune stromal elements, such as cancer-associated fibroblasts (CAFs), a major cellular component of tumour stroma that promotes tumorigenesis and cancer progression through paracrine signalling and modulation of the extracellular matrix. Neal et al. have used an air–liquid interface (ALI) organoid model that contains both integrated epithelial and stromal compartments to propagate patient-derived organoids (PDOs) from more than 100 biopsy samples as well as syngeneic mouse tumours^67^ (Fig. 2b). Interestingly, this study used a unique protocol to derive tumoroids while preserving the primary tumour epithelium *en bloc* with endogenous immune and non-immune stromal elements. The appropriateness of this model—termed ALI-PDO—was supported by a sustainable diversity of the immune repertoire (T cells, B cells, natural killer cells and macrophages) together with an integrated stroma expressing mesenchymal markers. More importantly, antibodies against programmed death-ligand 1 (PD-L1) or its receptor PD1 induced the efficient expansion and activation of TILs in such human- and mouse-derived organoids, which consequently elicited tumour cytotoxicity.^67^ In this context, this authentic in vitro model represents an ideal opportunity to investigate network complexity in response to ionising radiation on its own or in combination with immunotherapy.

In addition to the phenotypic observations such as cellular expansion or death, transcriptomics analysis of the different cellular components (tumour epithelial cells, CAFs and immune cells) after cell sorting would be extremely useful in informing how each cellular compartment behaves with or without radiation, and to measure the co-operative effects of immunotherapy in this system (Fig. 2b). It would also be helpful to profile the immune repertoire in order to gain insights into changes that might be induced by radiation alone and radiation combined with immunotherapy. In the meantime, time-of-flight mass cytometry (CyTOF) might be a useful technique to broadly interrogate the systemic response and the balance of cell subsets before and after treatment.^68,69^ Finally, this derived ALI-PDO system could serve as a platform for high-throughput drug screening (e.g. of existing and novel immunotherapeutics), as well as for experimental settings of the combination of ionising radiation and immunotherapy to determine the adequate regimen (dosage of immunotherapy, dose of radiation and timing of treatment) (Fig. 2b). It could be adapted for ‘in sitro’ technology, which originally consisted of the ‘in vitro’ and ‘in situ’ culture of oligocellular suspensions of fresh tumours upon the addition of chemotherapeutics. Thus, the measurements of hundreds of parameters of immune activation might become possible.^70^

## The abscopal effect

When it was first proposed in 1953, the term ‘abscopal’ referred to “an action at a distance from the irradiated volume but within the same organism”.^71^ Subsequently, from clinical experience and preclinical model systems, the ‘abscopal effect’ has been defined as a systemic consequence of radiation on ‘out-of-field’ tumour deposits that is mediated by local immune-effector cells and is capable of antitumour activity towards both targeted and distant lesions.^72^ The immunomodulatory properties of ionising radiation, therefore, offer novel avenues in the development of rational combination with immunotherapy. Such an approach is aimed at maximising local tumour control, eradicating existing metastases and inhibiting the potential for systemic metastases.^42^

The results of a seminal study, published in 2012, describe how treatment of metastatic melanoma with an immune-checkpoint inhibitor resulted in a complete resolution of the disease after palliative ionising radiation to a single lesion.^73^ Diverse immune-targeting strategies in combination with ionising radiation are currently under investigation in clinical trials in various tumour entities. These strategies include the co-administration of cytokines (e.g. IL-2, granulocyte–macrophage colony-stimulating factor [GM-CSF]), transfer of immune cells with antitumour activity (e.g. dendritic cells, cytokine-induced killer cells, lymphoid effectors and T lymphocytes with tumour-specific chimeric antigen receptors), gene-mediated cytotoxic immune therapy, vaccine therapy, immune-checkpoint inhibitors (e.g. anti-PD1, anti-PD-L1 and anti-CTLA-4), co-stimulatory agonists (e.g. CD137 and CD134) and Toll-like receptor agonists, as well as immunotherapy targeting the tumour microenvironment (e.g. tumour-associated macrophages or myeloid-derived cells).^74^ Discovery researchers and clinicians are therefore exploiting cutting-edge technologies in radiotherapy and cancer immunotherapy to further gain therapeutic efficiency.^75^ Herein, the complexity of radio-immunotherapy trials is evident, and the design of this research is mostly done using empirical considerations. Irradiation dose and fractionation regimen, as well as size, localisation and number of irradiated lesions are still key factors that remain to be characterised in greater detail. Moreover, such aspects must be coupled with immunotherapy to promote a high degree of synergy. We still know very little, and there are different ways to tackle this dilemma. Should we add immunotherapy to the standard of care based on radiation? Or, should we add radiation to the standard of care based on immunotherapy? Will we ultimately be able to exploit this synergy to make a rare abscopal effect a routine effect?

### What have we learnt so far from preclinical models about the radiotherapy–immunotherapy-induced abscopal effect?

Studies concerning irradiation-induced antitumour immunity and the abscopal effect in preclinical animal models are numerous. Each model per se is unique, with specific readouts, and uses either heterotopic transplantation or autochthonous tumours that are genetically engineered or induced by carcinogens. So far, preclinical models have taught us four lessons about antitumour immunity and its relationship with the abscopal effect. First, the occurrence of initial immune priming and subsequent immune-effector functions when a single tumour is irradiated. Second, the existence of a long-lasting systemic effect that occurs when previously irradiated animals are rechallenged with tumour cells, or naive animals were challenged after immune adoptive transfer from treated animals. Third, the observation of an abscopal effect on out-of-field lesions when regression was observed on the non-irradiated tumour in animals bearing two tumours or in half-irradiated organs harbouring nodules following injection of tumour cells. Fourth, the observation of an abscopal effect on distant metastases in autochthonous models.^76^ Consequently, these preclinical models have been used to study similar combinatorial strategies with immunotherapy, but their many inherent caveats, such as primary tumour location and treatment scheme applied to animals, mean that they do not mimic the clinical situation particularly closely. To better understand the molecular cues and the technical parameters leading to synergistic effects, it is necessary to rethink our experimental workflow in a more systematic manner to match preclinical models to clinical situations.

### Using tumour models to improve our knowledge

The tumour ecosystem in preclinical models differs from that in the clinical situation. In preclinical models, most tumours usually grow subcutaneously after injection of tumour cells and do not recapitulate the diversity of human tumours; indeed, these tumours are ‘unrealistic’ because a large amount of cancer cell death occurs shortly after injection, and because they grow rapidly in an ectopic environment in the absence of chronic inflammation.^70^ It is also important to note that skin layers are known to be highly populated with professional APCs, which might hinder the interpretation and extrapolation of preclinical findings to clinical practice.^77^ Based on these limitations, some authors have proposed generating a subcutaneous human tumour autograft, which would be considered as the primary tumour to be treated in order to prime a more efficient abscopic effect.^78^ This approach raises ethical concerns as well as safety and logistical issues, such as the source of cells and the site of engraftment, let alone the success of engraftment.

It could be argued that the benefits from the combined use of immunotherapy and ionising radiation are only observed in the skin location. To discard this hypothesis, it will be necessary to explore this dual strategy in models that are faithful to clinical situations, not restricted to skin tumours. The transgenic expression of oncogenes or inactivation of tumour-suppressor genes by genetic recombination results in the most powerful models of autochthonous cancers (Fig. 2c). For example, robust models of mammary or lung tumorigenesis originate from the MMTV–*Erbb2* transgenic cassette or through the induction of a specific *Kras* allele, respectively.^70^ Moreover, orthotopic models are also suitable because they closely resemble the natural tumorigenesis process.^79^ However, the use of autochthonous and orthotopic models is limited as the experimental handling is both time-consuming and cost-intensive (e.g. genetic manipulation, tumour development and tumour monitoring) when compared with transplantable tumours. To date, only a few experiments, investigating glioblastoma, pancreatic or breast cancers, using orthotopic syngeneic mouse models, have been described in the literature.^80–87^

Another technical limitation is the availability of irradiators for small animals to enable the precise irradiation of tumours arising in autochthonous and orthotopic models, as opposed to heterotopic tumours, which are located at the flank, footpad or thigh and are, therefore, more accessible for external radiotherapy. Advances in modern technologies using onboard-integrated image guidance might enable these constraints to be overcome,^88,89^ with the SARPP (for small-animal radiation research platform) (Xstrahl Ltd, UK)^90,91^ and the X-RAD 225CX (Precision X-ray Inc., USA)^92^ systems being extensively used in the field, especially for subcutaneously grown tumours, but they need to be adapted to these aforementioned tumour models. These two platforms offer a unique advantage, with different imaging modalities, including computed tomography, positron emission tomography or single photon emission tomography, magnetic resonance imaging and bioluminescence imaging.^89^ In addition to precise conformal radiotherapy dose distributions, they allow the real-time tracking of tumour cells in luciferase-engineered mouse models owing to bioluminescence capture. Zeng and colleagues have already exploited this technology to test the combination of anti-PD1 immunotherapy with stereotactic radiosurgery in an orthotopic glioblastoma mouse model.^81^ The authors showed that radiosurgery plus PD1 blockade generated a robust antitumour activity against primary intracranial gliomas, and that radiotherapy increased the pro-inflammatory profile of glioma cells. CD8 T-cell activity was then a marker of responsiveness to immunotherapies.^81^ Additional investigations involving these models are, therefore, required to functionally characterise the response to combined treatment with immunotherapy and ionising radiation. Furthermore, mass cytometry is indispensable for building a comprehensive overview of the global dynamics of immune cells, to ultimately leverage the effects with combined immunotherapy or to overcome induced resistance (Fig. 2c).

### Investigating the importance of tumour characteristics

In clinical situations, surgical debulking of the primary tumour and possibly its draining lymph nodes is usually undertaken before any other intervention, whereas in fully immune-competent rodents, primary tumours are generally first subjected to irradiation.^93^ However, the magnitude of the antitumour immune response to ionising radiation is known to be driven by the initial antigenic load liberated by damaged tumour cells in the irradiated field, as well as by the effective priming of T cells by APCs in lymph nodes.^94,95^ Moreover, the number and volume of irradiated lesions are also key parameters that could modulate treatment responses.^96,97^ Accordingly, setting up debulking surgery^98^ and removing lymph nodes^99^ in animal models are required to effectively investigate whether the primary tumour and lymph nodes are key facilitators of the abscopal effect. Even if primarily thought as dispensable, this experimental step is essential to prove whether the primary efficiency of the ionising radiation–immunotherapy combination partly relies on the bulk tumour and its draining nodes. Debulking surgery and lymph node removal might explain why the abscopal effect is far less likely to be observed in human tumours, but is systematically induced in rodents.

In terms of the number of lesions irradiated, the heterogeneity of most tumours should be taken into account, as TAAs released in response to irradiation of one lesion might not be representative of those of lesions in other anatomical locations, or might not be targeted by CTLs owing to localised immunosuppressive effects.^100,101^ Consequently, multisite radiotherapy, in association with immunotherapy, is expected to increase the exposure to TAAs—representative of the bulky disease—and to break down immunosuppressive barriers.^97^ The effects of single- lesion irradiation versus multisite-lesion irradiation in combination with immunotherapy should, therefore, be carried out in animal models with multiorgan metastasis. In this context, the 4T1 breast cancer cell line can give rise to orthotopic primary tumours and tumour metastases at multiple sites (lung, axillary nodes, brain and bone) in a syngeneic model.^102^ It would also be interesting to sequentially widen irradiation to various sites (e.g. primary tumour, metastatic lung, and bone) in order to investigate whether gross tumour irradiation and/or single or multiple lesions scale up tumour response, despite the obvious technical difficulties associated with this approach. Finally, it is also worth considering that chemotherapy might contribute to an increase in TAAs and consequently participate in the overall immunogenic effect.^103^ However, in contrast to this co-operative effect, chemotherapy might impede antitumour activity as a consequence of reduced cell viability and interference with T-cell functions as it occurs in clinical situations.^104–106^ Therefore, it is essential to implement models using autochthonous or orthotopic tumours that recapitulate the different therapeutic measures (i.e. chemotherapeutics + /− ionising radiation/immunotherapy) so as to evaluate the contribution of each modality in antitumour responses. Not only can the read-out of endpoints—in this case, decrease into tumour burden, absence of progressive disease and absence of relapse—inform on treatment efficacy, but high-throughput methods to study molecular and cellular aspects (e.g. gene expression and immune repertoire) are also crucially needed to assess treatment efficacy and to identify potential biomarkers that would be predictive of antitumour responses (Fig. 2c).

### Effective treatment planning

Treatment planning includes the irradiation regimen itself as well as the treatment sequence with immunotherapy. In terms of ionising radiation, varying doses and fractionations in combination with immunotherapy are areas that are undergoing active investigation. There have been conflicting preclinical results regarding whether a high-dose single-fraction approach is superior to a moderate- or low-dose, multiple-fraction approach. Some studies have supported the efficacy of a single dose ranging from 0.5 to 25 Gy, whereas others have demonstrated that fractionated doses of 2 Gy, or smaller hypofractionated doses of 6 or 8 Gy, were more efficient than a single larger dose.^107–111^ A comparative study between subablative hypofractionated radiation and ablative single-fraction stereotactic body radiotherapy identified a threshold dose of 10–12 Gy, above which radiation triggered immunosuppression and loss of the abscopal effect.^112^ So far, no consensus on the optimal dose-fractionation regimen has been reached. Furthermore, the effects of ionising radiation on the tumour microenvironment—immune infiltration and endothelial damage—also depend on the dose. Thus, a window of radiation dosing might be more effective for supporting tumour immunity.^109,113,114^

Clinical case reports have shown that radiotherapy has mostly been conducted as a palliative strategy, using a high-dose delivery.^73,115,116^ No clinical study has directly compared different dose-fraction regimens in conjunction with immunotherapy for the ability to trigger an abscopal response. Accordingly, more preclinical evidence is required to reveal a potential dose–response phenomenon and to determine the threshold above which ionising radiation plus immunotherapy is no longer efficient. In a model of subcutaneous tumours, Vanpouille-Box and colleagues elucidated the molecular mechanism by which an elevated dose of ionising radiation antagonised antitumour immunity.^112^ The DNA exonuclease three-prime-repair exonuclease 1 (TREX1) acts as a molecular sensor of cancer cells’ immunogenicity as its expression is induced upon high-dose irradiation, leading to clearance of double-stranded DNA in tumour cells, no IFN-β release by cancer cells and insufficient activation of antitumour response.^112^ Additional insights using suitable animal models, as previously proposed, should be then provided for establishing both an optimal dose-fractionation regimen and an optimal strategy for further clinical studies.

Regarding the treatment sequence of ionising radiation and immunotherapy, a few studies have evaluated the impact of the precise timing of immunotherapy administered with other cytotoxic approaches, and have revealed that the efficacy of immunotherapy depends on the timing of its administration, and is specific to its mechanisms of action.^87^ For example, in the case of an anti-CTLA-4 of specific isotype, administration before ionising radiation is optimal,^85,117^ whereas agents that facilitate checkpoint blockage (anti-CTLA-4 and anti-PD1/PD-L1), or agonists of cGAS-STING pathway should be administered shortly before and concurrently with ionising radiation,^85,86,110,111,118^ and co-stimulatory agents such as anti-OX40 are best administered rapidly after ionising radiation.^85^ Again, however, we lack extensive knowledge about these strategies in non-subcutaneous tumour models in order to confirm that both the optimal irradiation dose/fractionation and treatment sequencing are adopted for this approach to then be accurately translated into the clinic. It is also possible to derive upfront experimental specimens based on using the ALI-PDO model to probe the immune response following different schemes. This will enable a better understanding of when the synergistic mechanisms take place or fail to occur.^67^ Using this strategy, it is also tempting to test whether immunotherapy-based combinatorial approaches might be beneficial or detrimental in order to adjust an efficient immune response, with a precise timing of immunotherapy administration and dosing in view of radiotherapy (i.e. dose and fractionation) (Fig. 2c).

## Conclusion

**Fig. 3:**
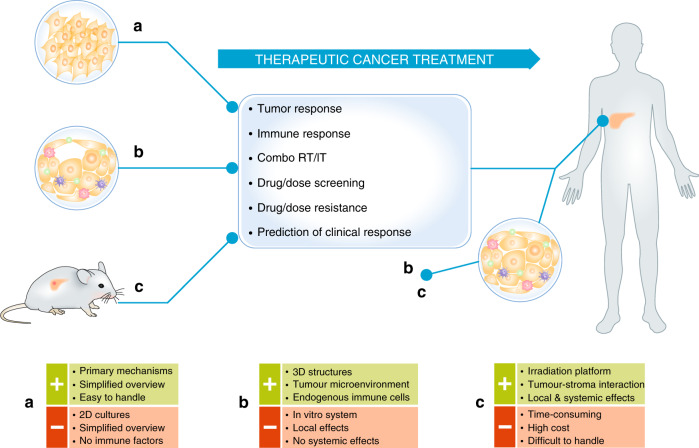
Applications of the 2D, 3D and mouse models in discovery cancer research and translational oncology. **a** Co-cultures, **b** mouse or patient-derived tumour organoids and **c** genetically engineered mouse models or patient-derived tumour xenografts. Advantages (+) and disadvantages (−) of each strategy are highlighted in green and red boxes, respectively. Human specimens might also serve as a study model using tumour organoids (condition (**b**)) or xenografts (condition (**c**)).

More than a century ago, Paul Ehrlich applied the term ‘Zauberkugel’ to describe “a drug specifically targeting a particular pathogen without affecting normal host cells”, inferring that “the optimal agent would combine high parasitotropism with low organotropism”.^119^ Since then, immunotherapies have emerged in the therapeutic arsenal as this so-called ‘magic bullet’, aimed at targeting specific antigens on cancer cells. As we constantly perceive the heterogeneity of tumours, and we understand the complexities of immunology, we might wonder how basic discoveries, translational insights and clinical studies will integrate over the next 20 years. Undoubtedly, cutting-edge technologies and next-generation drugs will turn the ‘magic bullet’ into a ‘silver bullet’ due to evidence-based consensus guidelines for radiation–immunotherapy combinations, as a perfect strategy that cures a disease with no danger of side effects. Despite some drawbacks, a combination of co-culture, 3D tumoroids and mouse models is indispensable to better understand the biology of non-targeted radiation effects. As previously mentioned, these strategies should be coupled to methods using human biopsy samples as models for large-scale screenings to ultimately improve current cancer treatments (Fig. 3). Multidisciplinary approaches are therefore required to unravel the immunological and genetic mechanisms to foster the efficacy of different therapies. Opportunities to deepen our basic knowledge about the bystander, cohort and abscopal effects of ionising radiation, and the use of ionising radiation in collaboration with cancer immunotherapy, await to be explored, with the ultimate aim of offering rational approaches to treat immune-mediated cancer as the next step in oncology practice.

## Data Availability

Not applicable.
